# Supplementary material for: Genetically engineered ESC-derived embryos reveal Vinculin-dependent force responses required for mammalian neural tube closure
Source: bioRxiv. 2025 Dec 25:2025.12.22.696028. Preprint. [Version 1] doi: 10.64898/2025.12.22.696028 (PMC12767355; doi:10.64898/2025.12.22.696028)
Supplement: 1 [file NIHPP2025.12.22.696028V1-supplement-1.pdf]

bioRxiv preprint doi: https://doi.org/10.64898/2025.12.22.696028; this version posted December 25, 2025. The copyright holder for this preprint (which was not certified by peer review) is the author/funder, who has granted bioRxiv a license to display the preprint in perpetuity. It is made available under aCC-BY 4.0 International license.

Figure 1—figure supplement 1. The response to edge ablations in the cranial neural plate requires actomyosin contractility

(A) Localization of the tight junction protein ZO-1 in the lateral midbrain of early and late elevation wild-type embryos (see Figure 1C and D for quantification). (B and C) ML edges before and 2-8 s after ablation in late elevation embryos expressing GFP-Plekha7 and treated for 2 h with water (B) or 200 µM of the Rho-kinase inhibitor Y-27632 (C). (D) Peak recoil velocity after laser ablation of ML edges. Boxes, 25th-75th percentile; whiskers, 5th-95th percentile; horizontal line, median; +, mean. 15 ablations in 4 embryos/condition. ****p<0.0001 (Welch's t-test). Maximum intensity projections, anterior up, edges oriented vertically in kymographs. Bars, 10 µm.

Figure 2—figure supplement 1. Generation and validation of *Shroom3* mutant and GFP-Plekha7 ESCs

(A and B) ESC clones homozygous for a null mutation in *Shroom3* (A) were identified by PCR screening and deep sequencing (B). (A) Red box, deleted region within exon 5 of the *Shroom3-203* isoform. (B) Deletion breakpoints of four homozygous mutant clones. Top, 5' and 3' gRNA target sites. Red, PAM site. Dotted lines, predicted CRISPR cut sites. Bottom, deletion breakpoints. (C) Shroom3 protein is not detected by immunofluorescence in the lateral midbrain in $Shroom3^{ESC}$ embryos. (D and E) Embryos generated by injection of GFP-Plekha7-expressing ESCs displayed GFP-Plekha7 expression throughout the cranial neural plate (D) (black regions, dividing cells) that colocalized with N-cadherin (E). Maximum intensity projections, anterior up. Bars, 100 µm (D), 10 µm (C and E). (F) Pups generated from the GFP-Plekha7-expressing ESC clone *R26-PHA7-EGFP A3*. 25/27 animals had 100% ESC coat color and 2/2 adults tested displayed germline transmission.

Figure 3—figure supplement 1. Generation and validation of *Vinculin* mutant ESCs

(A and B) ESC clones homozygous or heterozygous for a null mutation in *Vinculin* (A) were identified by PCR screening and deep sequencing (B). (A) Red box, deleted region including exon 3 of the *Vinculin-201* isoform. (B) Deletion breakpoints of *Vinculin* homozygous mutant clone 58 and heterozygous control clone 15. Top, 5' and 3' gRNA target sites. Red, PAM site. Dotted lines, predicted CRISPR cut sites. Bottom, deletion breakpoints. (C) Vinculin protein is absent in $Vinculin^{ESC}$ embryos (generated from clone 58) and present in $Control^{ESC}$ embryos (generated from clone 15) and +/+ embryos (FVB/N) (one E9.5 embryo/lane). (D) Light micrographs of E9.5 $Control^{ESC}$ and $Vinculin^{ESC}$ embryos (23/23 $Vinculin^{ESC}$ embryos and 0/15 $Control^{ESC}$ embryos displayed exencephaly). (E) Light micrographs of E9.5 wild-type and $Vinculin^{\Delta Epi}$ embryos (11/11 $Vinculin^{\Delta Epi}$ embryos and 0/30 wild-type and heterozygous littermate controls displayed exencephaly). Lateral views, dotted lines indicate the lateral edges of the cranial neural plate. Bars, 500 µm.

bioRxiv preprint doi: https://doi.org/10.64898/2025.12.22.696028; this version posted December 25, 2025. The copyright holder for this preprint (which was not certified by peer review) is the author/funder, who has granted bioRxiv a license to display the preprint in perpetuity. It is made available under aCC-BY 4.0 International license.

Figure 3—figure supplement 2. Apical-basal elongation, proliferation, and apoptosis are unaffected in *Vinculin* mutants

(A) Apical-basal cell height in transverse sections of the lateral midbrain of Control^{ESC} and *Vinculin*^{ESC} embryos in early elevation (4-6 somites), late elevation (7-9 somites), and apposition (8-10 somites). A single value was obtained for each embryo and the mean±SEM between embryos is shown (3-4 embryos/genotype). *p<0.03 (Welch's t-test). (B) Dividing cells detected with phospho-histone H3 in the lateral midbrain of wild-type and *Vinculin*$^{\Delta Epi}$ embryos in late elevation. Cell outlines are visualized with ZO-1. (C) Percentage of cells positive for phospho-histone H3 in wild-type and *Vinculin*$^{\Delta Epi}$ embryos. Boxes, 25th-75th percentile; whiskers, 5th-95th percentile; horizontal line, median; +, mean, 6-7 regions in 3-4 embryos/genotype. (D, E) Detection of the apoptotic cell marker cleaved caspase 3 is similar in wild-type and *Vinculin*$^{\Delta Epi}$ embryos in early (D) and late (E) elevation. Maximum intensity projections, anterior up. Bars, 10 µm (B), 100 µm (D, E).

Figure 4—figure supplement 1. Actomyosin localization in *Vinculin*^{ESC} and *Vinculin*$^{\Delta Epi}$ embryos

(A) Localization of myosin IIB in Control^{ESC} and *Vinculin*^{ESC} embryos in late elevation. Bottom panels, examples of multicellular junctions with wild-type myosin IIB localization (left) and moderate (cyan) or severe (red) gaps in myosin II localization (right). (B) Localization of F-actin (phalloidin) in Control^{ESC} and *Vinculin*^{ESC} embryos in late elevation. (C and D) Normalized intensity profiles of myosin IIB (C) and F-actin (phalloidin) (D) along 3 µm lines perpendicular to bicellular junctions. An average value was obtained for 10 bicellular junctions/neural fold and the mean±SEM between neural folds is shown (8 neural folds in 4 embryos/genotype). Maximum intensity projections, anterior up. Bars, 10 µm (A, top panels, B), 2 µm (A, bottom panels).

Figure 4—figure supplement 2. Generation and validation of *Vinculin* mutant ESCs expressing GFP-Plekha7

(A) Deletion breakpoints of *Vinculin* homozygous mutant clone 41 expressing GFP-Plekha7. Top, 5' and 3' gRNA target sites. Red, PAM site. Dotted lines, predicted CRISPR cut sites. Bottom, deletion breakpoints based on deep sequencing of amplicons around the 5' and 3' cut sites. (B) Vinculin protein is absent in *Vinculin*^{ESC} GFP-Plekha7 embryos (generated from clone 41) and present in Control^{ESC} GFP-Plekha7 embryos (generated from unedited wild-type GFP-Plekha7 ESCs) (one E9.5 embryo/lane). (C) Light micrographs of E9.5 Control^{ESC} GFP-Plekha7 and *Vinculin*^{ESC} GFP-Plekha7 embryos (28/28 *Vinculin*^{ESC} GFP-Plekha7 embryos and 0/30 Control^{ESC} GFP-Plekha7 embryos displayed exencephaly). Lateral views, dotted lines indicate the lateral edges of the cranial neural plate. Bar, 500 µm.

Figure 4—figure supplement 3. Quantification of GFP-Vinculin expression from the *R26* locus in ESC-derived embryos.

(A) Western blot showing the presence of GFP-Vinculin in addition to the endogenous Vinculin protein detected with the anti-Vinculin antibody (one embryo/lane). (B, C) Vinculin (B) and GFP-Vinculin (C) proteins showed no significant difference in total protein levels between 0-4 somite and 7-12 somite stages. Protein intensity was normalized to the intensity of the β-catenin loading control. Boxes, 25th-75th percentile; whiskers, 5th-95th percentile; horizontal line, median; +, mean.

bioRxiv preprint doi: https://doi.org/10.64898/2025.12.22.696028; this version posted December 25, 2025. The copyright holder for this preprint (which was not certified by peer review) is the author/funder, who has granted bioRxiv a license to display the preprint in perpetuity. It is made available under aCC-BY 4.0 International license.

Figure 5—figure supplement 1. *Vinculin$^{\Delta Epi}$* embryos display defects in F-actin and adherens junction localization.

(A and B) Number of gaps in N-cadherin localization at bicellular junctions in a 50 µm x 50 µm region of the lateral midbrain in Control^{ESC} and *Vinculin^{ESC}* embryos in early (A) and late (B) elevation. (C, D) Localization of N-cadherin in Control^{ESC} and *Vinculin^{ESC}* embryos in early and late elevation (reproduced from Figure 5A and B). Yellow circles show all tricellular and multicellular junctions scored as defective. (E) Localization of N-cadherin and F-actin (phalloidin) in wild-type and *Vinculin$^{\Delta Epi}$* embryos in late elevation. (F and G) Localization of GFP-Plekha7 and F-actin (phalloidin) (F) and number of gaps in GFP-Pha7 localization (G) in a 50 µm x 50 µm region of the lateral midbrain in wild-type and *Vinculin$^{\Delta Epi}$* embryos in late elevation. Arrowheads show examples of multicellular junctions scored as defective. 6 regions in 3 embryos/genotype in A, B, and G. Maximum intensity projections, anterior up. Bars, 10 µm.

Figure 6—figure supplement 1. Tight junction and adherens junction protein localization in *Vinculin^{ESC}* and *Vinculin$^{\Delta Epi}$* embryos

(A) Localization of the tight junction protein ZO-1 and the adherens junction protein GFP-Plekha7 in early elevation Control^{ESC} and *Vinculin^{ESC}* embryos. (B, C) Number (left) and areas (right) of adherens junction gaps (B) and tight junction gaps (C) in a 50 µm x 50 µm region in early elevation Control^{ESC} and *Vinculin^{ESC}* embryos. Note the differences in scale between the adherens junction and tight junction plots. Boxes, 25th-75th percentile; whiskers, 5th-95th percentile; horizontal line, median; +, mean. 38-106 gaps from 6 regions in 3 embryos. *p<0.03 (Welch's t-test). (D and E) Normalized intensity profiles of ZO-1, GFP-Plekha7, and myosin IIB measured along 3 µm lines parallel to the apical-basal axis of bicellular junctions in XZ reconstructions of AiryScan z-stacks in late elevation wild-type and *Vinculin$^{\Delta Epi}$* embryos. An average value was obtained for 10 bicellular junctions/neural fold and the mean±SEM between neural folds is shown (4 neural folds in 2 embryos/genotype). Maximum intensity projections, anterior up. Bar, 10 µm.

Figure 7—figure supplement 1. Apical constriction, high-order junctions, and cell division initiate correctly in *Vinculin^{ESC}* embryos

(A) Stills from time-lapse movies of Control^{ESC} (top) and *Vinculin^{ESC}* (bottom) embryos expressing GFP-Plekha7, color coded by apical cell area. (B) The average apical cell area decreases similarly in Control^{ESC} embryos and in *Vinculin^{ESC}* embryos that did not display severe defects in cell adhesion. n=4 Control^{ESC} embryos/time point, 5 *Vinculin^{ESC}* embryos at 0 h, 4 *Vinculin^{ESC}* embryos at 1.5 h, and 2 *Vinculin^{ESC}* embryos at 3.0 h. (C, D) The numbers of rosettes (C) and dividing cells (D) were not significantly different in Control^{ESC} and *Vinculin^{ESC}* embryos. (E) Division times (time elapsed between the onset of cleavage furrow ingression and the appearance of GFP-Plekha7 at the new vertex or interface) in Control^{ESC} (top) and *Vinculin^{ESC}* (bottom) embryos. A single value was obtained for each embryo and the mean±SEM between embryos is shown in (B). Boxes, 25th-75th percentile; whiskers, 5th-95th percentile; horizontal line, median; +, mean. 4-5 embryos/genotype in (C-E). Maximum intensity projections, anterior up. Bars, 10 µm.

bioRxiv preprint doi: https://doi.org/10.64898/2025.12.22.696028; this version posted December 25, 2025. The copyright holder for this preprint (which was not certified by peer review) is the author/funder, who has granted bioRxiv a license to display the preprint in perpetuity. It is made available under aCC-BY 4.0 International license.

Supplementary File 1. N values and details of statistical analyses performed.

Supplementary Movie 1. Time-lapse movie of a Control^{ESC} embryo expressing GFP-Plekha7. Images were acquired every 6 min for 3 hr. Maximum intensity projections, anterior up. Bar, 10 μm.

Supplementary Movie 2. Time-lapse movie of a moderately defective *Vinculin^{ESC}* embryo expressing GFP-Plekha7. Images were acquired every 6 min for 3 hr. Maximum intensity projections, anterior up. Bar, 10 μm.

Supplementary Movie 3. Time-lapse movie of a severely defective *Vinculin^{ESC}* embryo expressing GFP-Plekha7. Images were acquired every 6 min for 3 hr. Maximum intensity projections, anterior up. Bar, 10 μm.

bioRxiv preprint doi: https://doi.org/10.64898/2025.12.22.696028; this version posted December 25, 2025. The copyright holder for this preprint (which was not certified by peer review) is the author/funder, who has granted bioRxiv a license to display the preprint in perpetuity. It is made available under aCC-BY 4.0 International license.

Figure 1—figure supplement 1

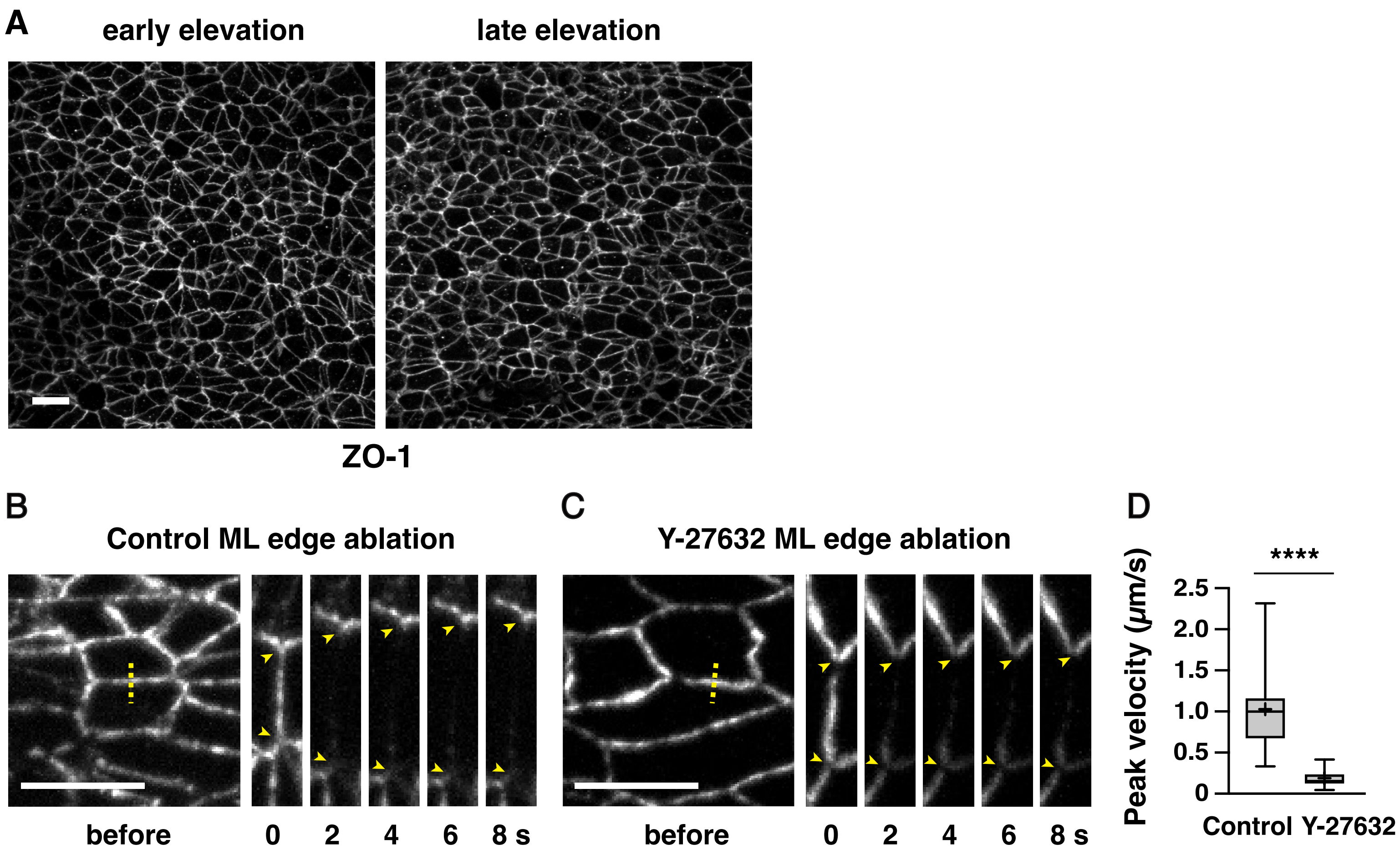

Figure 2—figure supplement 1

bioRxiv preprint doi: https://doi.org/10.64898/2025.12.22.696028; this version posted December 25, 2025. The copyright holder for this preprint (which was not certified by peer review) is the author/funder, who has granted bioRxiv a license to display the preprint in perpetuity. It is made available under aCC-BY 4.0 International license.

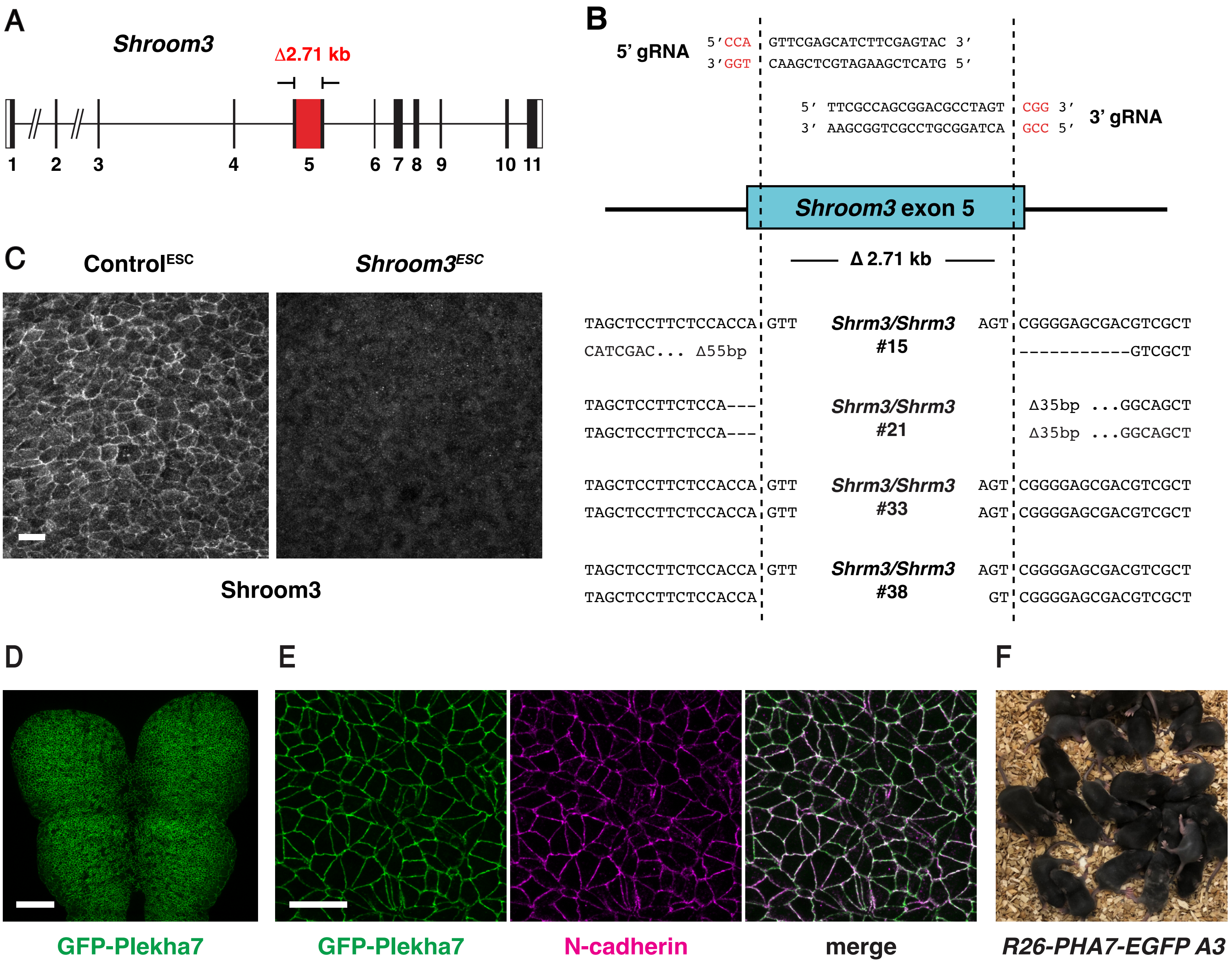

bioRxiv preprint doi: https://doi.org/10.64898/2025.12.22.696028; this version posted December 25, 2025. The copyright holder for this preprint (which was not certified by peer review) is the author/funder, who has granted bioRxiv a license to display the preprint in perpetuity. It is made available under aCC-BY 4.0 International license.

Figure 3—figure supplement 1

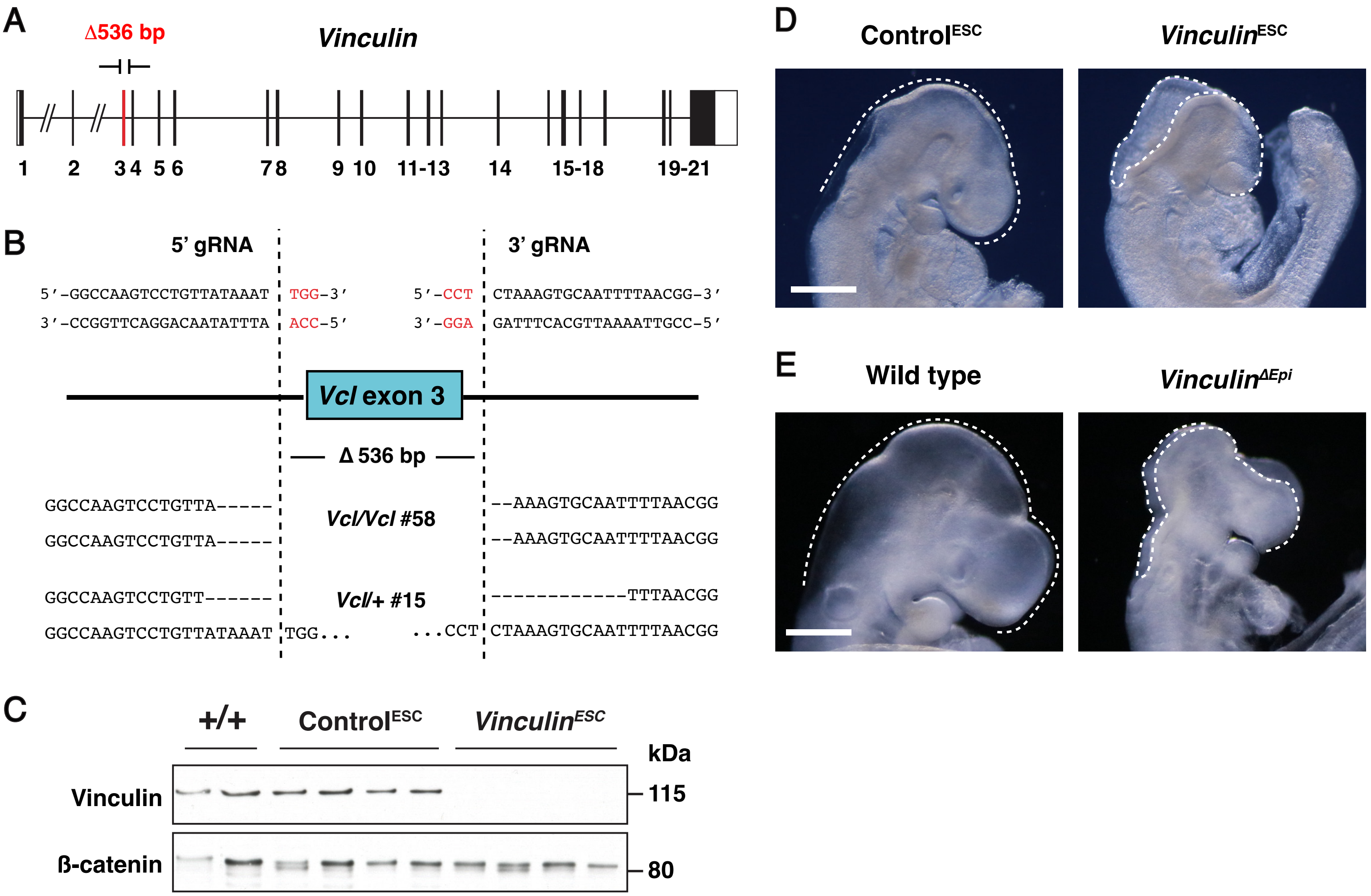

bioRxiv preprint doi: https://doi.org/10.64898/2025.12.22.696028; this version posted December 25, 2025. The copyright holder for this preprint (which was not certified by peer review) is the author/funder, who has granted bioRxiv a license to display the preprint in perpetuity. It is made available under aCC-BY 4.0 International license.

Figure 3—figure supplement 2

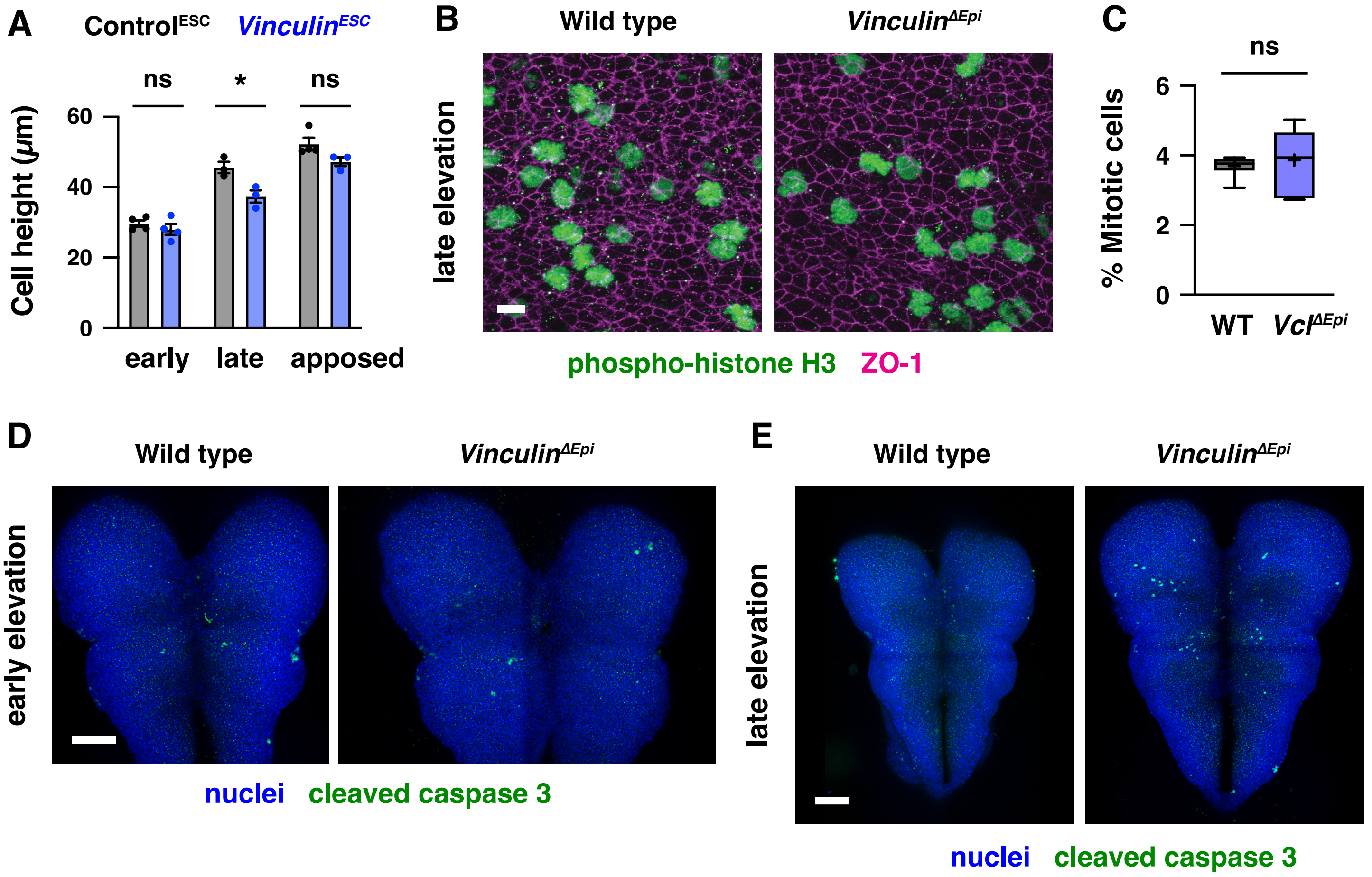

Figure 4—figure supplement 1

bioRxiv preprint doi: https://doi.org/10.64898/2025.12.22.696028; this version posted December 25, 2025. The copyright holder for this preprint (which was not certified by peer review) is the author/funder, who has granted bioRxiv a license to display the preprint in perpetuity. It is made available under aCC-BY 4.0 International license.

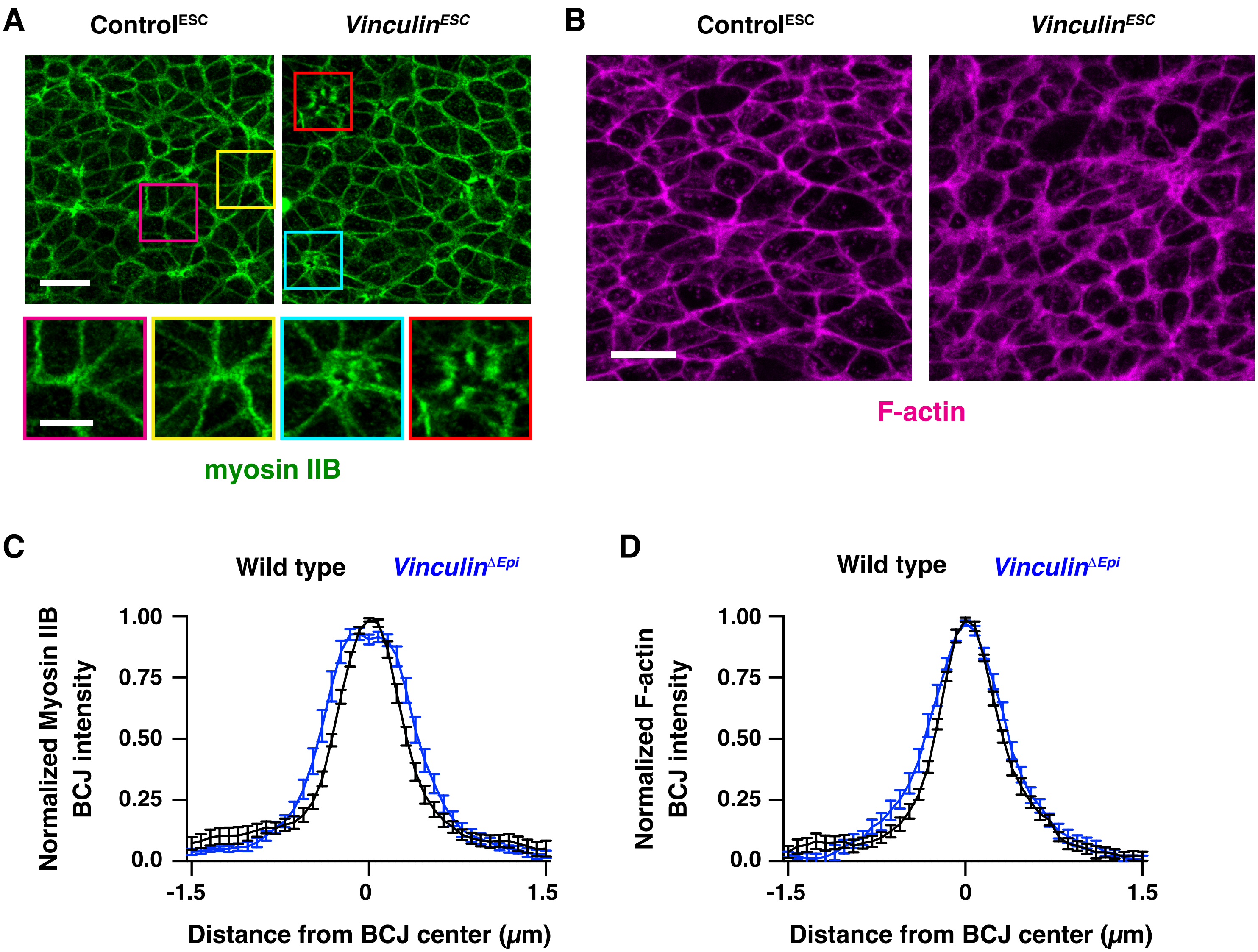

bioRxiv preprint doi: https://doi.org/10.64898/2025.12.22.696028; this version posted December 25, 2025. The copyright holder for this preprint (which was not certified by peer review) is the author/funder, who has granted bioRxiv a license to display the preprint in perpetuity. It is made available under aCC-BY 4.0 International license.

Figure 4—figure supplement 2

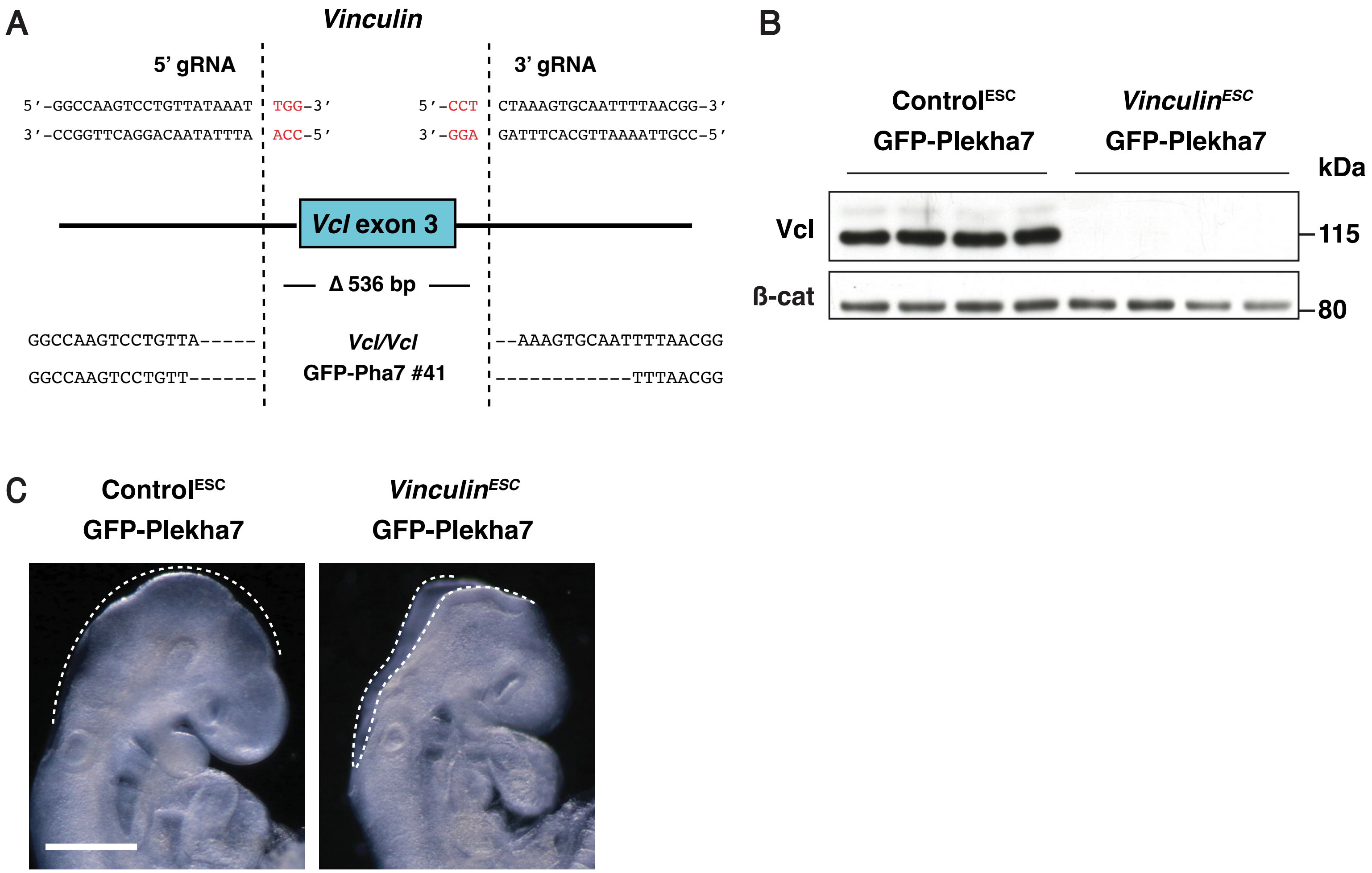

Figure 4—figure supplement 3

bioRxiv preprint doi: https://doi.org/10.64898/2025.12.22.696028; this version posted December 25, 2025. The copyright holder for this preprint (which was not certified by peer review) is the author/funder, who has granted bioRxiv a license to display the preprint in perpetuity. It is made available under aCC-BY 4.0 International license.

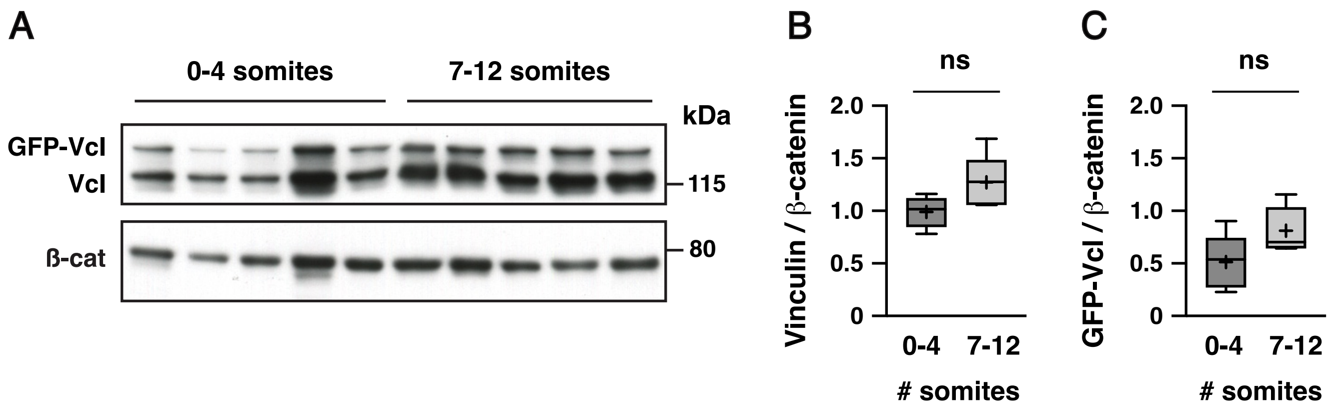

Figure 5—figure supplement 1

bioRxiv preprint doi: https://doi.org/10.64898/2025.12.22.696028; this version posted December 25, 2025. The copyright holder for this preprint (which was not certified by peer review) is the author/funder, who has granted bioRxiv a license to display the preprint in perpetuity. It is made available under aCC-BY 4.0 International license.

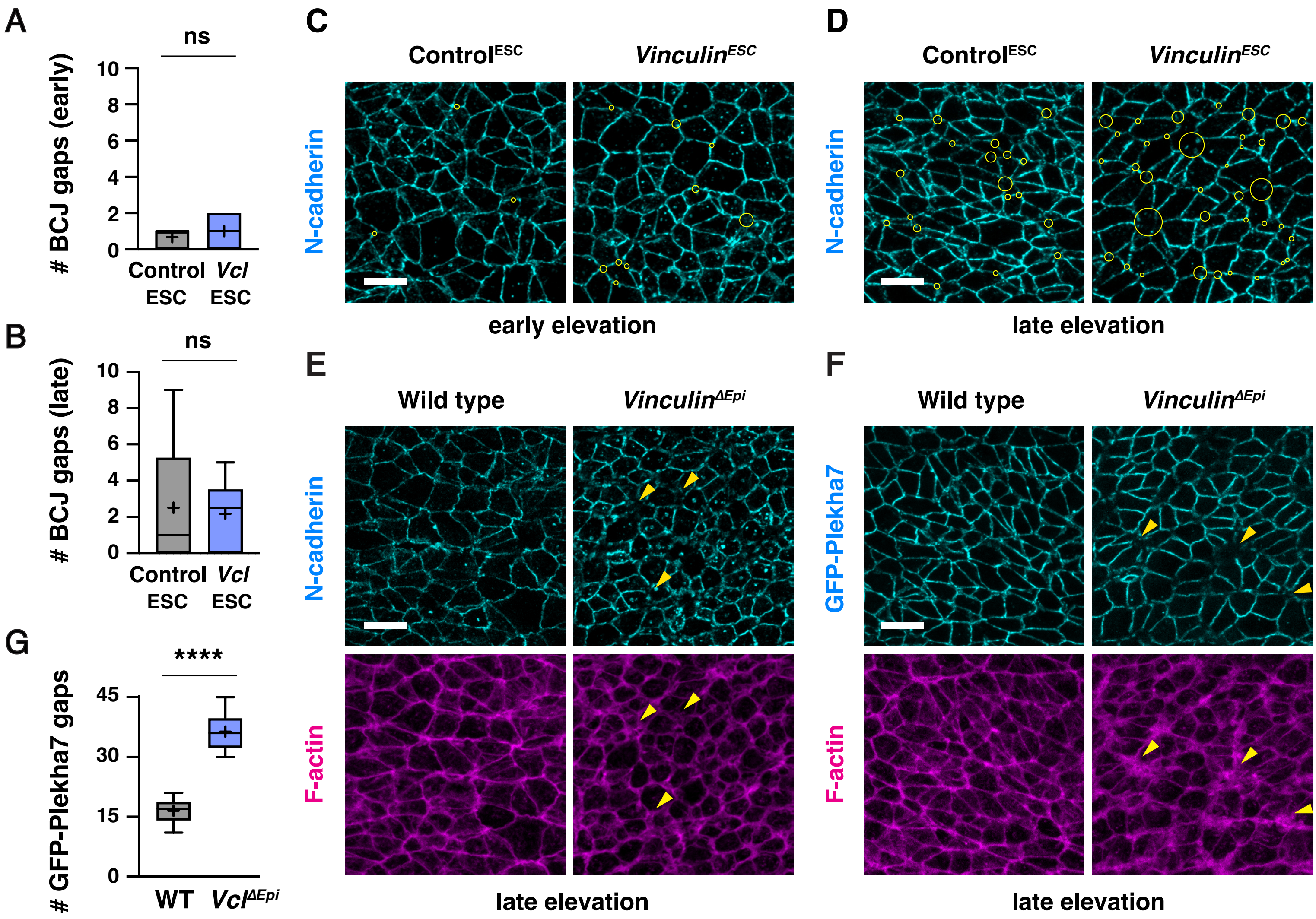

bioRxiv preprint doi: https://doi.org/10.64898/2025.12.22.696028; this version posted December 25, 2025. The copyright holder for this preprint (which was not certified by peer review) is the author/funder, who has granted bioRxiv a license to display the preprint in perpetuity. It is made available under aCC-BY 4.0 International license.

Figure 6—figure supplement 1

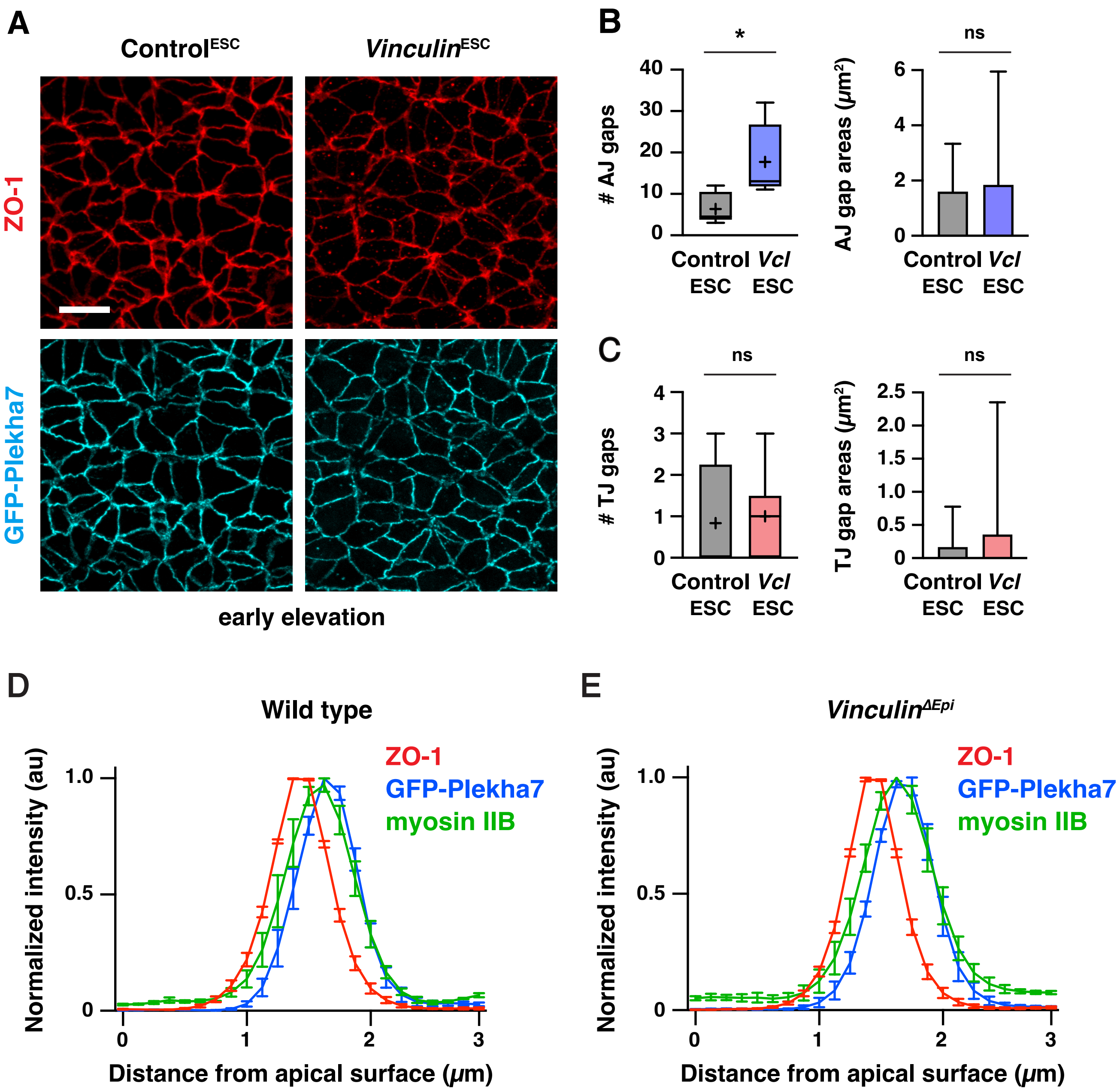

bioRxiv preprint doi: https://doi.org/10.64898/2025.12.22.696028; this version posted December 25, 2025. The copyright holder for this preprint (which was not certified by peer review) is the author/funder, who has granted bioRxiv a license to display the preprint in perpetuity. It is made available under aCC-BY 4.0 International license.

Figure 7—figure supplement 1

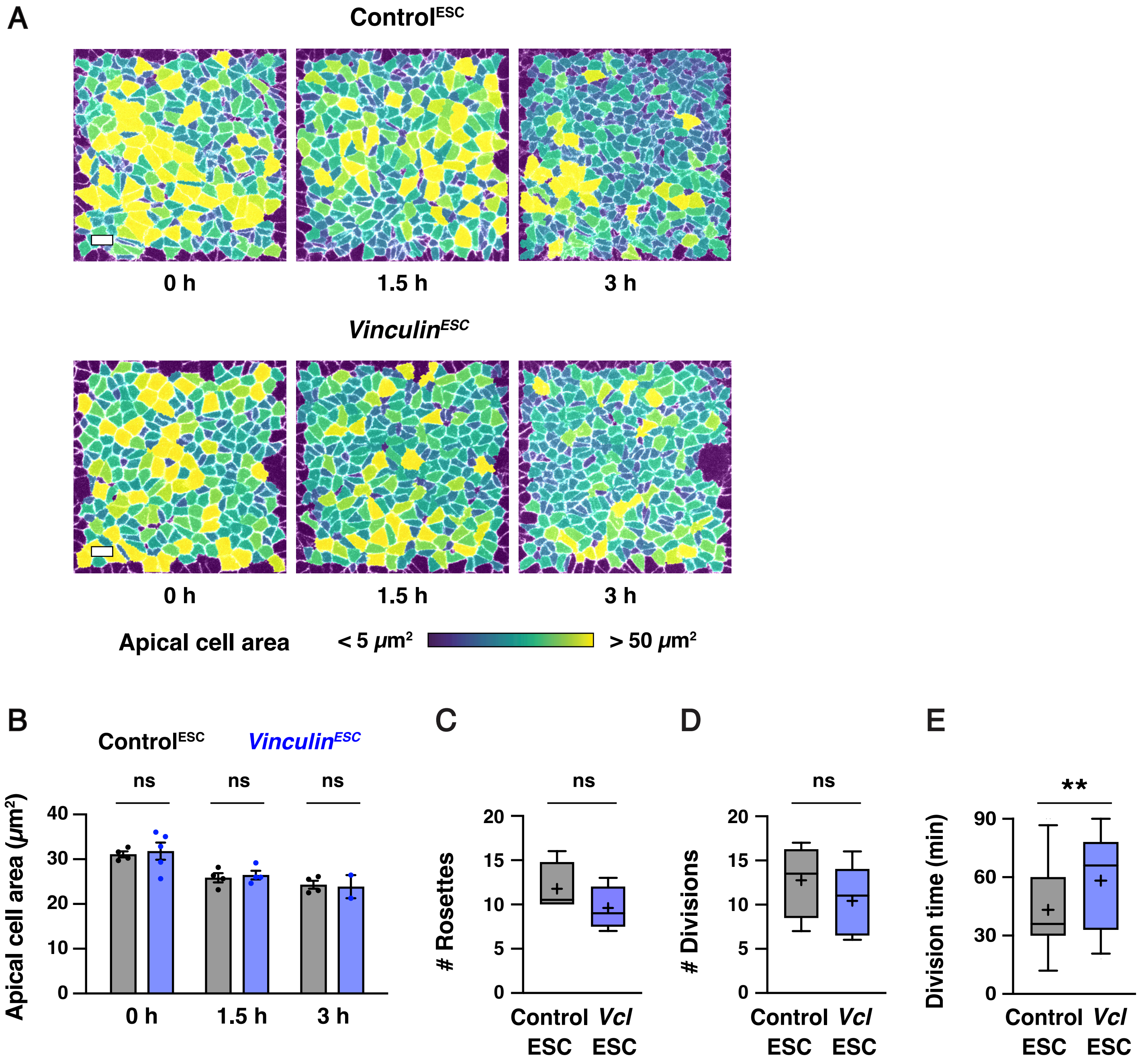

bioRxiv preprint doi: https://doi.org/10.64898/2025.12.22.696028; this version posted December 25, 2025. The copyright holder for this preprint (which was not certified by peer review) is the author/funder, who has granted bioRxiv a license to display the preprint in perpetuity. It is made available under aCC-BY 4.0 International license.

Supplementary File 1. N values and details of statistical analyses performed.

Figure	Condition	n values	Mean ± SEM (unless noted)	P value	Statistical test
1C	ZO-1 early	8 regions in 4 emb	1.02±0.04	0.31 (ZO-1 vs MyoIIB)<br><br>0.076 (ZO-1 vs pMRLC)	Welch's t-test
	Myo IIB early		1.10±0.06		
	pMRLC early		1.19±0.08		
1D	ZO-1 late	8 regions in 4 emb	1.02±0.03	0.24 (ZO-1 vs MyoIIB)<br><br>0.011 (ZO-1 vs pMRLC)	Welch's t-test
	Myo IIB late		1.10±0.06		
	pMRLC late		1.40±0.11		
1H, L	ML early	18 ablations in 9 emb	0.58±0.05	0.0002 (ML early vs ML late)<br><br>0.033 (AP early vs AP late)<br><br>0.006 (ML early vs AP early)<br><br>0.002 (ML late vs AP late)	Welch's t-test
	ML late	19 ablations in 6 emb	1.29±0.15		
	AP early	16 ablations in 8 emb	0.38±0.04		
	AP late	17 ablations in 7 emb	0.65±0.11		
2E	Ctl^{ESC}	3 emb (912, 740, 1092 cells/emb)	20.05±2.18	0.011	Welch's t-test
	Shroom3^{ESC}	3 emb (545, 484, 543 cells/emb)	33.65±1.26		
2F	Ctl^{ESC}	2744 cells in 3 emb	N/A	<0.0001	Kolmogorov-Smirnov test
	Shroom3^{ESC}	1572 cells in 3 emb			
3C	Ctl^{ESC} early	4 emb	1.19±0.04	0.66	Welch's t-test
	Vcl^{ESC} early	4 emb	1.17±0.02		
	Ctl^{ESC} late	3 emb	0.95±0.01	0.0035	
	Vcl^{ESC} late	3 emb	1.15±0.02		
	Ctl^{ESC} apposed	4 emb	0.76±0.03	0.0009	
	Vcl^{ESC} apposed	3 emb	1.07±0.01		
3E	Ctl^{ESC} pre-elevation	4 emb (505, 465, 413, 484 cells/emb)	36.34±1.43	0.63	Welch's t-test
	Vcl^{ESC} pre-elevation	3 emb (395, 435, 505 cells/emb)	37.91±2.59		
	Ctl^{ESC} early	3 emb (614, 654, 599 cells/emb)	27.78±0.78	0.55	
	Vcl^{ESC} early	3 emb (631, 641, 513 cells/emb)	29.29±2.06		
	Ctl^{ESC} late	4 emb (912, 1173, 1088, 782 cells/emb)	18.08±1.53	0.036	
	Vcl^{ESC} late	4 emb (726, 830, 799, 664 cells/emb)	23.48±1.26		
3F	Ctl^{ESC} pre-elevation	1867 cells in 4 emb	N/A	0.0090	Kolmogorov-Smirnov test
	Vcl^{ESC} pre-elevation	1335 cells in 3 emb			

bioRxiv preprint doi: https://doi.org/10.64898/2025.12.22.696028; this version posted December 25, 2025. The copyright holder for this preprint (which was not certified by peer review) is the author/funder, who has granted bioRxiv a license to display the preprint in perpetuity. It is made available under aCC-BY 4.0 International license.

3G	Ctl^{ESC} early	1867 cells in 3 emb	N/A	<0.0001	Kolmogorov-Smirnov test
	Vcl^{ESC} early	1785 cells in 3 emb			
3H	Ctl^{ESC} late	3955 cells in 4 emb	N/A	<0.0001	Kolmogorov-Smirnov test
	Vcl^{ESC} late	3019 cells in 4 emb			
4E	Ctl^{ESC} early	17 ablations in 5 emb	0.68±0.12	0.50	Welch's t-test
	Vcl^{ESC} early	26 ablations in 5 emb	0.59±0.07		
4F	Ctl^{ESC} late	14 ablations in 7 emb	1.19±0.20	0.31	Welch's t-test
	Vcl^{ESC} late	15 ablations in 5 emb	1.44±0.14		
4H	GFP-Pha7 early	60 TCJs in 3 emb	1.40±0.04	<0.0001	Welch's t-test
	GFP-Vcl early	80 TCJs in 4 emb	3.65±0.25		
4I	GFP-Pha7 late	60 TCJs in 3 emb	1.06±0.04	<0.0001	Welch's t-test
	GFP-Vcl late	80 TCJs in 4 emb	1.82±0.07		
4K	early	80 TCJs in 4 emb	58.7±2.5	<0.0001	Welch's t-test
	late	80 TCJs in 4 emb	72.4±2.3		
5E	Ctl^{ESC} early	6 regions in 3 emb	4.00±1.67 (mean±SD)	0.0015	Welch's t-test
	Vcl^{ESC} early	5 regions in 3 emb	11.60±2.79 (mean±SD)		
5F	Ctl^{ESC} late	6 regions in 3 emb	18.17±4.83 (mean±SD)	<0.0001	Welch's t-test
	Vcl^{ESC} late	6 regions in 3 emb	34.83±3.97 (mean±SD)		
5G	Ctl^{ESC} TCJ	142-175 TCJs/region 8 regions in 4 emb	4.9±0.6	<0.0001	Welch's t-test
	Vcl^{ESC} TCJ	81-153 TCJs/region 12 regions in 6 emb	17.7±1.3		
5H	Ctl^{ESC} 4CJ	36-57 4CJs/region 8 regions in 4 emb	14.1±1.0	<0.0001	Welch's t-test
	Vcl^{ESC} 4CJ	21-36 4CJs/region 12 regions in 6 emb	48.6±3.4		
5I	Ctl^{ESC} 5+ CJ	11-19 5+ CJs/region 8 regions in 4 emb	31.6±3.7	<0.0001	Welch's t-test
	Vcl^{ESC} 5+ CJ	7-17 5+ CJs/region 12 regions in 6 emb	77.8±3.6		
6B	Ctl^{ESC} (AJ gap #)	8 regions in 4 emb	18.75±2.12 (mean±SD)	<0.0001	Welch's t-test
	Vcl^{ESC} (AJ gap #)	12 regions in 6 emb	41.50±7.94 (mean±SD)		
	Ctl^{ESC} (AJ gap area)	150 gaps in 4 emb	0.97±0.95 (mean±SD)	<0.0001	
	Vcl^{ESC} (AJ gap area)	498 gaps in 6 emb	3.23±6.60 (mean±SD)		
6C	Ctl^{ESC} (TJ gap #)	8 regions in 4 emb	1.13±1.13 (mean±SD)	0.0004	Welch's t-test
	Vcl^{ESC} (TJ gap #)	12 regions in 6 emb	4.68±2.39 (mean±SD)		
	Ctl^{ESC} (TJ gap area)	150 gaps in 4 emb	0.03±0.13 (mean±SD)	<0.0001	
	Vcl^{ESC} (TJ gap area)	498 gaps in 6 emb	0.64±2.44 (mean±SD)		

bioRxiv preprint doi: https://doi.org/10.64898/2025.12.22.696028; this version posted December 25, 2025. The copyright holder for this preprint (which was not certified by peer review) is the author/funder, who has granted bioRxiv a license to display the preprint in perpetuity. It is made available under aCC-BY 4.0 International license.

7D	Ctl^{ESC} (no gap)	47 rosettes in 4 emb	89.4±7.1	0.0007 (no gap)<br>0.017 (gap repairs)<br>0.65 (gap persists)<br>0.11 (gap expands)	Welch's t-test
	Ctl^{ESC} (gap repairs)		1.6±1.6		
	Ctl^{ESC} (gap persists)		9.1±7.1		
	Ctl^{ESC} (gap expands)		0.0±0.0		
	Vcl^{ESC} (no gap)	48 rosettes in 5 emb	29.1±7.6		
	Vcl^{ESC} (gap repairs)		44.9±11.1		
	Vcl^{ESC} (gap persists)		13.0±3.9		
	Vcl^{ESC} (gap expands)		13.1±6.4		
	Ctl^{ESC} (% defective junctions)	47 rosettes in 4 emb	10.6±7.1	0.0007	
	Vcl^{ESC} (% defective junctions)	48 rosettes in 5 emb	70.9±7.6		
7E	Ctl^{ESC} (neighbor contacts)	51 divisions in 4 emb	8.0±4.7	0.0008	Welch's t-test
	Vcl^{ESC} (neighbor contacts)	52 divisions in 5 emb	73.3±9.2		
	Ctl^{ESC} (new contacts)	51 divisions in 4 emb	1.5±1.5	0.013	
	Vcl^{ESC} (new contacts)	52 divisions in 5 emb	48.9±11.3		
7F	Ctl^{ESC}	50 divisions in 4 emb	N/A	0.021	Kolmogorov-Smirnov test
	Vcl^{ESC}	28 divisions in 5 emb			

1 S1D	Control	15 ablations in 4 emb	1.03±0.15	<0.0001	Welch's t-test
	Y-27632	15 ablations in 4 emb	0.19±0.03		
3 S2A	Ctl^{ESC} early	4 emb	29.64±0.93	0.40	Welch's t-test
	Vcl^{ESC} early	4 emb	27.98±1.54		
	Ctl^{ESC} late	3 emb	45.62±1.59	0.026	
	Vcl^{ESC} late	3 emb	37.35±1.77		
	Ctl^{ESC} apposed	4 emb	52.29±1.76	0.069	
	Vcl^{ESC} apposed	3 emb	47.27±1.27		
3 S2C	WT	6 regions in 3 emb	3.7±0.1	0.64	Welch's t-test
	$Vcl^{\Delta Epi}$	7 regions in 4 emb	3.9±0.3		
4 S3B	0-4 somites	5 emb	0.99±0.07	0.076	Welch's t-test
	7-12 somites	5 emb	1.27±0.11		
4 S3C	0-4 somites	5 emb	0.51±0.12	0.089	Welch's t-test
	7-12 somites	5 emb	0.81±0.10		

bioRxiv preprint doi: https://doi.org/10.64898/2025.12.22.696028; this version posted December 25, 2025. The copyright holder for this preprint (which was not certified by peer review) is the author/funder, who has granted bioRxiv a license to display the preprint in perpetuity. It is made available under aCC-BY 4.0 International license.

5 S1A	Ctl^{ESC}	6 regions in 3 emb	0.67±0.21	0.53	Welch's t-test
	Vcl^{ESC}	5 regions in 3 emb	1.00±0.45		
5 S1B	Ctl^{ESC}	6 regions in 3 emb	2.50±1.43	0.84	Welch's t-test
	Vcl^{ESC}	6 regions in 3 emb	2.17±0.79		
5 S1G	WT	6 regions in 3 emb	16.50±3.39 (mean±SD)	<0.0001	Welch's t-test
	Vcl$^{\Delta Epi}$	6 regions in 3 emb	36.33±5.13 (mean±SD)		
6 S1B	Ctl^{ESC} (AJ gap #)	6 regions in 3 emb	6.33±3.72 (mean±SD)	0.023	Welch's t-test
	Vcl^{ESC} (AJ gap #)	6 regions in 3 emb	17.67±8.71 (mean±SD)		
	Ctl^{ESC} (AJ gap area)	38 gaps in 3 emb	1.60±1.73 (SD)	0.62	
	Vcl^{ESC} (AJ gap area)	106 gaps in 3 emb	1.85±4.10 (SD)		
6 S1C	Ctl^{ESC} (TJ gap #)	6 regions in 3 emb	0.83±1.33 (SD)	0.82	Welch's t-test
	Vcl^{ESC} (TJ gap #)	6 regions in 3 emb	1.00±1.10 (SD)		
	Ctl^{ESC} (TJ gap area)	38 gaps in 3 emb	0.17±0.10 (SD)	0.38	
	Vcl^{ESC} (TJ gap area)	106 gaps in 3 emb	0.36±1.99 (SD)		
7 S1B	Ctl^{ESC} (t = 0 h)	4 emb (300, 272, 261, 275 cells/emb)	31.07±0.67	0.74	Welch's t-test
	Vcl^{ESC} (t = 0 h)	5 emb (236, 254, 326, 280, 242 cells/emb)	31.79±1.92		
	Ctl^{ESC} (t = 1.5 h)	4 emb (388, 330, 311, 345 cells/emb)	25.84±1.04	0.68	
	Vcl^{ESC} (t = 1.5 h)	4 emb (318, 293, 334, 311 cells/emb)	26.45±0.97		
	Ctl^{ESC} (t = 3 h)	4 emb (401, 350, 332, 393 cells/emb)	24.29±0.89	0.89	
	Vcl^{ESC} (t = 3 h)	2 emb (318, 393 cells/emb)	23.86±2.59		
7 S1C	Ctl^{ESC}	47 rosettes in 4 emb	11.75±1.44	0.28	Welch's t-test
	Vcl^{ESC}	48 rosettes in 5 emb	9.60±1.08		
7 S1D	Ctl^{ESC}	51 divisions in 4 emb	12.75±2.10	0.43	Welch's t-test
	Vcl^{ESC}	52 divisions in 5 emb	10.40±1.81		
7 S1E	Ctl^{ESC}	50 divisions in 4 emb	43.20±2.96	0.007	Welch's t-test
	Vcl^{ESC}	28 divisions in 5 emb	58.28±4.50		
